# Prevalence and factors associated with intimate partner violence experiences among young married women in Nigeria: a cross-sectional study

**DOI:** 10.3389/fgwh.2026.1807793

**Published:** 2026-06-19

**Authors:** Aanuoluwapo Afolabi, Olayinka Ilesanmi

**Affiliations:** 1 MSI Nigeria Reproductive Choices, Abuja, Nigeria; Global Health and Infectious Diseases Institute, Nasarawa State University with MSI Nigeria Reproductive Choices, Abuja, Nigeria; 2 Global Health and Infectious Diseases Institute, Nasarawa State University, Keffi, Nigeria; 3 West Africa Regional Coordinating Centre, Africa Centre for Disease Control, Abuja, Nigeria

**Keywords:** autonomy, intimate partner violence, Nigeria, reproductive health, young women

## Abstract

In Nigeria, where early marriage and entrenched gender norms persist, evidence on intimate partner violence (IPV) among young married women remains limited and often narrowly focused on age-related risk. This study aimed to examine the prevalence of IPV and factors associated with IPV experiences among young married women in selected states in Nigeria. This household cross-sectional study was conducted among 872 young married women aged 15–24 years in Niger, Ondo, and Oyo States using an interviewer-administered questionnaire. IPV was defined as any experience of emotional, physical, or sexual violence perpetrated by a husband. Bivariate associations were assessed using chi-square tests, and multivariate logistic regression was used to determine factors independently associated with IPV at a 5% significance level. Overall, 26.5% of respondents reported experiencing IPV, with emotional violence being the most common form. In adjusted analyses, women with primary or no formal education (AOR = 4.28; 95% CI: 1.93–9.50) and secondary education (AOR = 2.51; 95% CI: 1.25–5.05) had significantly higher odds of IPV compared with those with tertiary education. Residence in rural (AOR = 2.53; 95% CI: 1.43–4.50) and urban areas (AOR = 3.36; 95% CI: 1.92–5.87) was associated with increased likelihood of experiencing IPV relative to peri-urban residence. High autonomy in household decision-making was also associated with higher odds of IPV (AOR = 2.32; 95% CI: 1.42–3.80). These findings emphasize the need for strengthened primary prevention strategies that address structural and sociocultural drivers of IPV among young married women. The Federal and State Ministries of Health, in collaboration with community stakeholder, should prioritize interventions that promote educational attainment, transform harmful gender norms, engage men and community leaders, and implement community-based behavior change programs targeting high-risk residential settings. While integrating routine IPV screening and referral into youth-friendly services remains important, greater emphasis must be placed on upstream interventions aimed at preventing IPV before it occurs.

## 1 Introduction

Intimate partner violence (IPV), defined as physical, psychological, or sexual harm perpetrated by a current or former intimate partner, constitutes a major global public health and human rights concern (1, 2). IPV has well-established consequences for women's physical, sexual, and mental health, including injury, unintended pregnancy, sexually transmitted infections, depression, and reduced utilization of health services (3–5). Beyond individual health outcomes, IPV constrains women's autonomy and participation in social and economic life, generating substantial costs for households, communities, and health systems (6). Contemporary public health frameworks conceptualize IPV not as isolated interpersonal behavior, but as an outcome shaped by the interaction of individual characteristics, relationship dynamics, and broader structural and gendered power relations (7, 8). This perspective is grounded in the ecological model of violence, which posits that IPV arises from interacting influences at multiple levels; individual, relational, community, and societal, and has been widely applied in violence research (9).

Globally, IPV is overwhelmingly perpetrated by male intimate partners (3). An estimated one in three women worldwide has experienced physical and/or sexual violence by an intimate partner in her lifetime (10). Adolescents and young women experience disproportionately high levels of IPV, reflecting heightened vulnerability during early adulthood and the initial years of marriage (11). From a life course and ecological perspective, early marriage may coincide with limited educational attainment, economic dependence, and reduced decision-making power, factors that increase susceptibility to violence within intimate unions (12). Although approximately 30% of girls aged 15–19 years and a similar proportion of women aged 20–24 years report experiencing IPV (13), young married women warrant distinct examination from a life-course and gender–power perspective, as early marriage situates them at a critical developmental transition marked by pronounced age and economic asymmetry between them and their husbands, constrained bargaining power, and limited social capital; conditions that may uniquely shape both their vulnerability to IPV and their capacity to respond to it. Within the ecological framework, these factors operate across multiple levels, including individual (e.g., age, education), relational (e.g., spousal age differences), and structural (e.g., norms supporting early marriage), reinforcing cumulative risk.

The burden of IPV is particularly pronounced in sub-Saharan Africa, where prevalence remains high across diverse socio-cultural settings (14). Nigeria, the most populous country in the region, represents a critical context for examining IPV among young married women (14). Structural theories of gender inequality highlight how entrenched patriarchal norms, early marriage, and unequal access to education and economic opportunities can reinforce power asymmetries within intimate relationships (15, 16). Within such contexts, relationship-level factors, such as age differentials between spouses, economic dependence, and male partner behaviors, may interact with individual socio-demographic characteristics to shape women's risk of experiencing IPV (15, 16). Women's autonomy in household decision-making and their ability to negotiate reproductive choices, including contraceptive use, are frequently conceptualized as observable dimensions of relational power within intimate unions. From a gender–power perspective, constrained reproductive agency and limited decision-making authority may both reflect and reinforce unequal power structures that heighten vulnerability to IPV. Conversely, shifts in women's autonomy within inequitable gender systems may also provoke backlash, further underscoring the complex relationship between empowerment and violence (16, 17). In parallel, the gender and power framework, as articulated by Connell (18, 19), provides a theoretical basis for understanding how socially structured inequalities, manifested through the sexual division of labor, sexual division of power, and norms regulating gender relations, shape women's vulnerability to IPV. Altogether, these dimensions align with the gender–power framework, in which constraints on women's agency reflect structural imbalances in power and access to resources.

Despite increasing policy attention to gender-based violence in Nigeria, including national legal reforms, important gaps remain in population-based evidence on IPV among adolescent and young married women. Nigeria's large youth population, high rates of early marriage in several regions, entrenched gender norms, and marked rural–urban disparities create a context in which early marital power imbalances may uniquely shape IPV risk. Yet much of the existing literature treats women as a homogeneous adult population, with limited attention to younger married cohorts or to the multilevel determinants operating during early marriage. Guided explicitly by the ecological model (9) and the gender and power framework (18, 19), quantitative analyses that examine individual, relational, and contextual correlates of IPV within this subgroup are essential for identifying modifiable risk factors and informing targeted primary prevention strategies. This study aimed to determine the prevalence and factors associated with IPV experiences among young married women in Nigeria.

## 2 Methods

### 2.1 Study design

This was a quantitative cross-sectional study conducted between January and March 2025.

### 2.2 Study area

This study was conducted in Niger, Ondo, and Oyo States in Nigeria, representing diverse socio-cultural, demographic, and geographic contexts across the North-Central and South-West regions. Nigeria operates a federal system comprising 36 states and 774 Local Government Areas (LGAs), with considerable heterogeneity in levels of urbanization, educational attainment, reproductive health indicators, and gender norms across regions.

Niger State, located in the North-Central zone, is the largest state in Nigeria by landmass and consists of 25 LGAs. The state is predominantly rural, with dispersed agrarian settlements and relatively lower levels of female education and reproductive health service utilization compared with southern regions of the country. Contraceptive prevalence in Niger State is lower than national and South-West averages, and women's participation in household and health-related decision-making remains comparatively limited (16). Early marriage is more common in parts of the state, contributing to age and economic asymmetries within unions.

Ondo and Oyo States are in the South-West geopolitical zone and comprise 18 and 33 LGAs respectively. These states are more urbanized and economically diversified, with major metropolitan centers such as Akure (Ondo State) and Ibadan (Oyo State), alongside peri-urban and rural agricultural communities. Although contraceptive prevalence in the South-West is comparatively higher than in North-Central Nigeria, substantial unmet need for family planning persists, particularly for birth spacing (16). Across these states, women's autonomy in health-related decision-making and negotiation of contraceptive use varies across rural, peri-urban, and urban contexts. In all three states, documented patterns of marital control behaviors, including jealousy, accusations of infidelity, and restrictions on women's mobility, remain prevalent, reflecting enduring gender norms that shape relationship dynamics.

To capture this socio-spatial diversity, three LGAs were selected in each state, one predominantly urban, one peri-urban, and one predominantly rural LGA. Within each selected LGA, three political wards were randomly sampled to ensure representation of communities across the urban–rural continuum. The inclusion of states from two geopolitical zones and varying levels of urbanization provides an opportunity to examine IPV within diverse socio-cultural and structural environments.

### 2.3 Study population

This study was conducted among women aged 15–24 years who have been married for at least one year in Niger, Ondo, and Oyo States; thus, the unit of enquiry was the household. Young married women aged 15–24 years represent one of the most vulnerable yet consistently overlooked populations in IPV research (10). To compute the sample size, we used the formula for cross-sectional studies, 50% prevalence, non-response rate of 10%, and a design effect of 2.0. Overall, the minimum sample size estimated was 854; however, the total number of respondents interviewed was 872.

### 2.4 Sampling procedure and technique

A multistage sampling design was employed to obtain a geographically diverse and contextually representative sample of young married women aged 15–24 years across selected states in Nigeria. The sampling approach was structured to capture variation across urban, peri-urban, and rural settings within each state. In each of the three study states (Niger, Ondo, and Oyo), three Local Government Areas (LGAs) were selected to reflect settlement diversity: one predominantly urban LGA, one peri-urban LGA, and one predominantly rural LGA. Within each selected LGA, three political wards were selected using simple random sampling techniques from the official list of wards. Subsequently, one community was selected using simple random sampling technique from each ward.

Within selected communities, a household listing was conducted to identify households with at least one eligible respondent. Households were then selected using simple random sampling from the compiled list of eligible households. Eligible participants were young married women aged 15–24 years who were usual residents of the household at the time of the survey. Where more than one eligible respondent was identified within a household, one participant was selected using simple random selection to avoid intra-household clustering.

### 2.5 Instrument

Data were collected using an electronic questionnaire adapted from existing literature including the Demographic Health Survey questionnaire (17). The questionnaire had three sections, including:

1. Sociodemographic characteristics (covering respondents' age, highest level of education, employment status, number of children, residence, husband's education and employment status, and household possession)
2. Woman's involvement in household decision making: This spanned questions such as woman's involvement in decisions about her own healthcare, her involvement in decisions about large and daily household purchases, her involvement in decisions about daily household meals, her involvement in decisions on how to spend her earnings and husband's earnings, and her involvement in decisions about the number of children to have in the marriage.
3. Pattern of IPV experienced: Prevalence of physical, sexual, and emotional violence was assessed using direct and clearly phrased questions asking respondents about their personal experiences of specific abusive acts. Physical violence was measured by asking whether a current or former partner had ever engaged in behaviours such as slapping or throwing objects capable of causing harm, pushing or shoving, hitting with a fist or other object, kicking, dragging or beating, deliberately choking or burning, or threatening or using a weapon such as a gun or knife. Sexual violence was assessed by asking women whether they had ever been physically forced to have sexual intercourse against their will, had sexual intercourse out of fear of what their partner might do, or had been compelled to engage in sexual acts they found degrading or humiliating.

Emotional violence captured non-physical forms of abuse. Respondents were asked whether a current or former partner had ever engaged in behaviours intended to demean, intimidate, or control them emotionally. These included being insulted or made to feel bad about themselves, being humiliated or belittled in front of others, being threatened with harm to themselves or someone close to them and being intentionally intimidated through actions such as yelling or aggressive behaviour. Consistent with the NDHS approach (16), a woman was considered to have experienced emotional violence if she reported at least one of these listed behaviours.

### 2.6 Interviewer selection and training

To encourage openness and ensure participant safety, interviews were conducted by 12 female data collectors and supervisors who were carefully selected based on emotional maturity, empathy, and their ability to interact respectfully with individuals from diverse backgrounds. Interviewers received comprehensive, standardized training aligned with World Health Organization guidance, including completion of the WHO Abortion Care Values Clarification and Attitudinal Transformation course. This training focused on ethical research practices, survivor-centred interviewing, confidentiality, and appropriate responses to sensitive disclosures, thereby enhancing the quality and integrity of the data collected.

### 2.7 Data collection

Interviews were conducted in private settings to ensure confidentiality and participant safety. In instances where respondents recalled or disclosed experiences of violence, research assistants demonstrated empathy and provided immediate emotional support. Where necessary, participants were offered counselling and referred to appropriate support services, including Sexual Assault Referral Centres or community-based organizations providing IPV-related services. Participation was voluntary, and informed consent was obtained from all respondents prior to data collection. Completed questionnaires were reviewed daily by field supervisors for completeness and consistency, and data were securely stored to protect participants' privacy. These procedures were implemented to ensure high-quality data collection while prioritizing the safety, dignity, and well-being of respondents.

### 2.8 Data analysis

Quantitative data was analyzed using STATA version 17. Descriptive statistics were summarized using frequencies and percentages. Participants' occupations were grouped into five categories: Professional/Technical/Managerial (higher education or leadership roles), Sales and Service (trading and service provision), Skilled Manual (artisanal and trade-based work), Others (miscellaneous occupations), and Unemployed (not working at the time of the study).

Autonomy in this study was defined as a woman's involvement in household decision-making across eight areas: her own health care, large household purchases, daily household needs, visits to relatives, food preparation, use of her own earnings, use of her husband's earnings, and decisions about the number of children. Each response was scored, with total possible scores ranging from 8 to 24. Using the mean cut-off of 16 points, women were grouped into three categories, namely: low autonomy (<16 points), medium autonomy (16–17 points), and high autonomy (>17 points).

IPV was defined as the experience of any emotional, physical, or sexual abuse. Emotional IPV included psychological abuse and economic control, sexual IPV encompassed coerced or forced sexual acts, and physical IPV covered direct harm or retaliatory aggression. A respondent was considered to have experienced IPV if she answered “Yes” to at least one indicator in any of the categories of IPV.

Chi-square tests were used to determine the association between IPV and respondents' sociodemographic characteristics. Variables that were significant at the bivariate level were pooled into the binary logistic regression model to control for confounders and determine the true factors associated with IPV among the young married women. The level of statistical significance was set below 5%.

### 2.9 Ethics approval

This study was approved by the Health Research Ethics Committees of the Oyo State Ministry of Health, Federal Medical Centre, Owo, Ondo State, and Federal Medical Centre, Bida, Niger State, Nigeria. Informed consent was obtained from each eligible participant prior to administering the questionnaire. Respondents were informed that they could exit the interview at any time. Respondents were not intentionally exposed to any form of harm due to this study.

## 3 Results

Overall, the mean age of the 872 young women was 21.3 ± 2.1 years; 16.6% were <20 years, 32.1% were aged 20–21 years, and 51.3% were ≥22 years. In total, 56.1% young women have had <3 children, 30.5% had 3–4 children, and 13.4% had >4 children. Also, 25.6% young women had primary or no formal education, 42.4% had secondary education, and 32.0% had tertiary education. Additionally, 11.7% husbands of young women had primary or no formal education, 42.8% had secondary education, and 45.5% had tertiary education. Overall, 22.4% lived in rural areas, 42.8% lived in urban, and 34.9% young women lived in peri-urban areas (Table 1).

**Table 1:** Sociodemographic characteristics of young married women in selected states in Nigeria.

Sociodemographic characteristics	Total = 872	Percentage
Age (Years): Mean = 2.1.3 ± 2.1		
18–19	145	16.6
20–21	280	32.1
≥22	447	51.3
Number of children born: Mean** =** 2.6 ± 1.7		
<3	489	56.1
3–4	266	30.5
>4	117	13.4
Woman's level of education		
Primary or none	223	25.6
Secondary	370	42.4
Tertiary	279	32.0
Employment status		
Professional/Technical/Managerial	112	12.8
Sales and service	332	38.1
Skilled manual	158	18.1
Other	80	9.2
Unemployed	190	21.8
Husband's level of education		
Primary or none	102	11.7
Secondary	373	42.8
Tertiary	397	45.5
Husband's employment status		
Professional/Technical/Managerial	252	28.9
Sales and service	207	23.7
Skilled manual	197	22.6
Other	160	18.3
Unemployed	56	6.4
Household wealth status		
Poorest	330	37.8
Poorer	160	18.3
Average	160	18.3
Richer	126	14.4
Richest	96	11.0
Place lived in		
Rural	195	22.4
Urban	373	42.8
Peri-urban	304	34.9

Table 2 describes the pattern of women's involvement in household decision making. In all, 56.8% young women jointly decided with their husbands about the women's healthcare, 21.9% made such decisions alone, the husbands of 18.6% decided alone, and 1.9% women decided about their healthcare with other persons (not husbands). Also, 50.7% young women jointly decided with their husbands on large household purchases, 32.3% women reported that their husbands made the decision on large household purchases alone, 14.9% women reported that they made the decision on large household purchases alone, and 0.9% women decided about large household purchases with other persons (not husbands).

**Table 2:** Involvement in household decision making among young married women across selected states in Nigeria.

Characteristics	Total
Person who usually decides on respondent's healthcare in this marriage	
Husband/Partner only	162 (18.6)
Respondent only	191 (21.9)
Respondent and husband	495 (56.8)
Respondent and another person	17 (1.9)
Someone else	7 (0.8)
Person who usually decides on large household purchases in this marriage	
Husband/Partner only	282 (32.3)
Respondent only	130 (14.9)
Respondent and husband	442 (50.7)
Respondent and another person	10 (1.1)
Someone else	8 (0.9)
Person who usually decides on household purchases for daily needs in this marriage	
Husband/Partner only	196 (22.5)
Respondent only	237 (27.2)
Respondent and husband	419 (48.1)
Respondent and another person	13 (1.5)
Someone else	7 (0.8)
Person who decides on visits to family or relatives in this marriage	
Husband/Partner only	150 (17.2)
Respondent only	247 (28.3)
Respondent and husband	458 (52.5)
Respondent and another person	12 (1.4)
Someone else	5 (0.6)
Person who usually decides about daily household meals in this marriage	
Husband/Partner only	168 (19.3)
Respondent only	389 (44.6)
Respondent and husband	296 (33.9)
Respondent and another person	15 (1.7)
Someone else	4 (0.5)
Person who usually decides how to spend respondent's own earnings in this marriage	
Husband/Partner only	38 (4.4)
Respondent only	544 (62.4)
Respondent and husband	283 (32.5)
Respondent and another person	6 (0.7)
Someone else	1 (0.1)
Person who usually decides what to do with money husband earns in this marriage	
Husband/Partner only	446 (51.1)
Respondent only	35 (4.1)
Respondent and husband	381 (43.7)
Respondent and another person	5 (0.6)
Someone else	2 (0.2)
Husband has no earning	2 (0.2)
Person who usually decides how many children respondent has in this marriage	
Husband/Partner only	91 (10.4)
Respondent only	136 (15.6)
Respondent and husband	640 (73.4)
Respondent and another person	5 (0.6)

**Figure 1:**
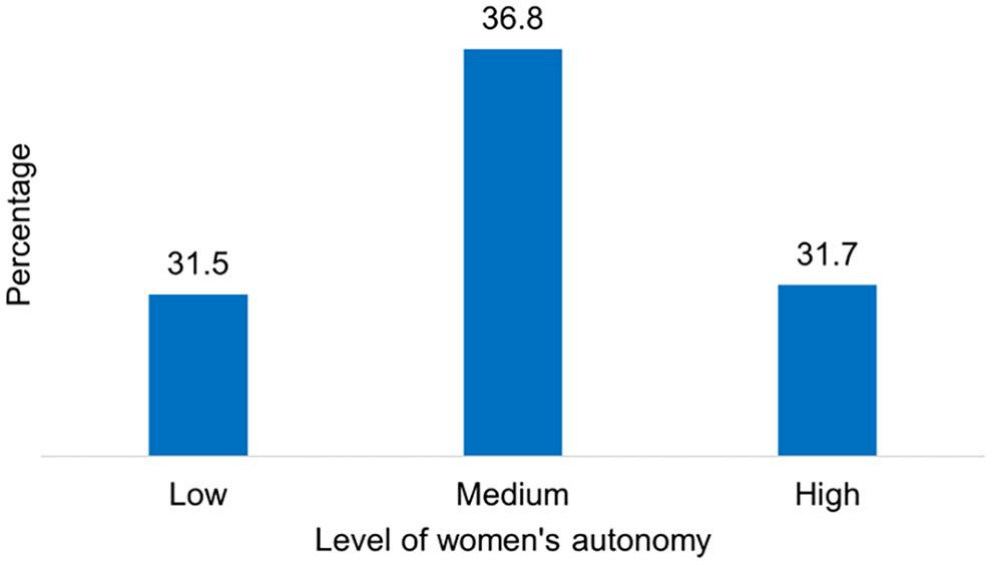
Autonomy in household decision making among young married woman in selected states in Nigeria.

In total, 31.5% young women had low autonomy in household decision making, 36.8% had medium autonomy, and 31.7% had high autonomy in household decision making (Figure 1).

Table 3 shows the pattern of IPV experienced by young women in selected states in Nigeria. In all, 2.1% young women had been physically forced by their husbands to have sexual intercourse when they did not want to. Also, 4.6% young women had been slapped or had something thrown at them by their husbands, which could hurt them. Likewise, 15.2% young women had been denied money for housekeeping or care of the children, and 13.1% young women had been denied money for their healthcare or other personal needs.

**Table 3:** Pattern of intimate partner violence experienced by young married women in selected states in Nigeria.

Characteristics	Frequency (%)
EMOTIONAL VIOLENCE	
Husband ignores her or treats her indifferently	
Yes	45 (5.2)
No	827 (94.8)
Ever been belittled by husband or made her feel she was not the woman he should have married	
Yes	23 (2.6)
No	849 (97.4)
Ever been expected by husband to take permission before woman seeks healthcare for herself	
Yes	131 (15.0)
No	741 (85.0)
Ever been forced to allow husband take all the important decisions in the house	
Yes	196 (22.5)
No	676 (77.5)
Ever been publicly humiliated or belittled by husband	
Yes	27 (3.1)
No	845 (96.9)
Ever been expected by husband to submit whenever he wants sex	
Yes	157 (18.0)
No	715 (82.0)
Ever had things done by husband to scare or intimidate her on purpose	
Yes	11 (1.3)
No	861 (98.7)
Husband ever told her that she is not good enough for him	
Yes	12 (1.4)
No	860 (98.6)
Ever been threatened by husband that he would hurt her or someone she cared about	
Yes	12 (1.4)
No	860 (98.6)
PHYSICAL VIOLENCE	
Ever got slapped or had something thrown at her by husband which could hurt her	
Yes	39 (95.5)
No	833 (95.5)
Ever been denied money for housekeeping or care of the children by husband	
Yes	108 (12.4)
No	764 (87.6)
Ever been denied money for her healthcare or other personal needs by husband	
Yes	83 (9.5)
No	789 (90.5)
Ever had her property of economic value seized by husband	
Yes	3 (0.3)
No	869 (99.7)
Ever threatened to be thrown out of the house or actually thrown out by husband	
Yes	13 (1.5)
No	859 (98.5)
Ever had her children or someone close to her beaten by husband in retaliation for what he thought she has done badly	
Yes	8 (0.9)
No	864 (99.1)
SEXUAL VIOLENCE	
Ever been physically forced by husband to have sexual intercourse when she did not want to	
Yes	20 (2.3)
No	852 (97.7)
Ever had sexual intercourse when she did not want it, but had it because she was afraid of what the husband might do	
Yes	6 (0.7)
No	866 (99.3)
Ever been forced by husband to do something she found degrading or humiliating	
Yes	10 (1.1)
No	862 (98.9)
Ever been forced by husband with threats or in any other way to perform sexual acts when she did not want to	
Yes	7 (0.8)
No	865 (99.2)
Ever been raped by husband	
Yes	8 (0.9)
No	864 (99.1)

**Figure 2:**
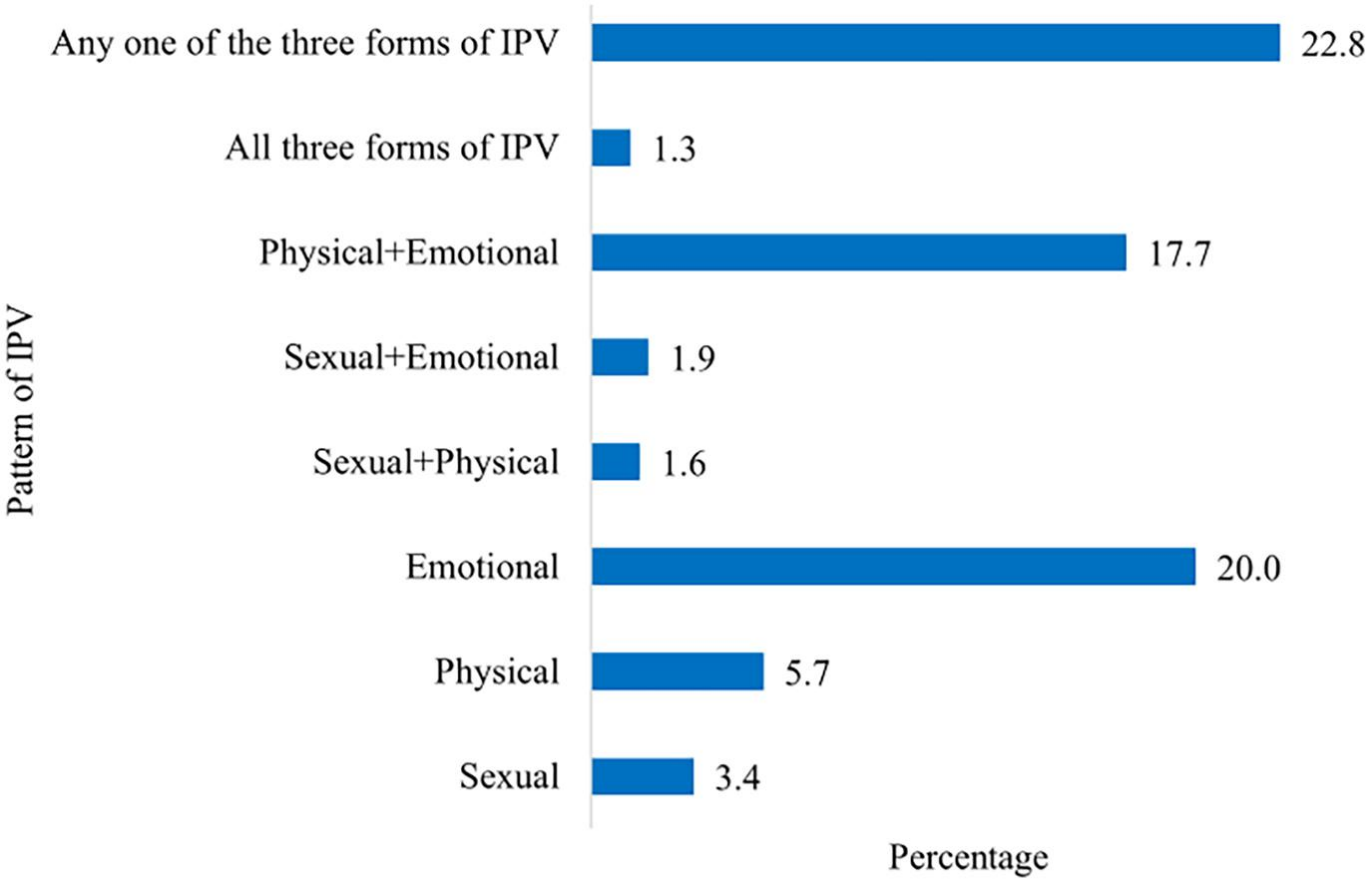
Pattern of intimate partner violence experienced by young married woman in selected states in Nigeria.

In all, 3.2% young women had experienced sexual violence from their husbands, 5.7% had experienced physical violence, 20.0% had experienced emotional violence from their husbands. Also, 1.6% young women had experienced sexual and physical violence, 1.9% had experienced sexual and emotional violence and 17.7% had experienced physical and emotional violence from their husbands. Overall, 1.3% young women had experienced all three forms of IPV from their husbands, while 22.8% had experienced any one of sexual, emotional, or physical violence from their husbands (*n* = 199) (Figure 2).

Table 4 shows the association between sociodemographic characteristics of young women and IPV experiences from their husbands. Overall, 32.4% women who got married at ages below 20 years had experienced IPV compared to 23.9% aged 20–21 years at marriage, and 19.0% aged 22 years or more at marriage (*p* ≤ 0.001). Also, 36.3% young women with primary or no formal education had experienced IPV compared to 24.1% with secondary education and 10.4% young women that have completed tertiary education (*p* ≤ 0.001). Likewise, 18.2% young women with low autonomy had experienced IPV compared to 14.3% with medium autonomy, and 37.3% young women with high autonomy (*p* ≤ 0.001).

**Table 4:** Association between intimate partner violence experiences and sociodemographic characteristics of young married women across selected states in Nigeria.

Characteristics	Experienced Intimate Partner Violence		Chi-square	*p*-value
	Yes	No		
	*n* (%)	*n* (%)		
Age (Years)				
<20	47 (32.4)	98 (67.6)	11.446	**<0** **.** **001**
20–21	67 (23.9)	213 (76.1)		
≥22	85 (19.0)	362 (81.0)		
Number of children born				
<3	107 (21.9)	382 (78.1)	3.162	0.206
3–4	70 (26.3)	196 (73.7)		
>4	22 (18.8)	95 (81.2)		
Woman's level of education				
Primary or none	81 (36.3)	142 (63.7)	47.862	**<0** **.** **001**
Secondary	89 (24.1)	281 (75.9)		
Tertiary	29 (10.4)	250 (89.6)		
Employment status				
Professional/Technical/Managerial	14 (12.5)	98 (87.5)	35.895	**<0** **.** **001**
Sales and service	74 (22.3)	258 (77.7)		
Skilled manual	23 (14.6)	135 (85.4)		
Other	17 (21.3)	63 (78.8)		
Unemployed	71 (37.4)	119 (62.6)		
Husband's level of education				
Primary or none	40 (39.2)	62 (60.8)	47.817	**<0** **.** **001**
Secondary	109 (29.2)	264 (70.8)		
Tertiary	50 (12.6)	347 (87.4)		
Husband's employment status				
Professional/Technical/Managerial	35 (13.9)	217 (86.1)	22.575	**<0** **.** **001**
Sales and service	64 (30.9)	143 (69.1)		
Skilled manual	55 (27.9)	142 (72.1)		
Other	34 (21.3)	126 (78.8)		
Unemployed	11 (19.6)	45 (80.4)		
Household wealth status				
Poorest	48 (14.5)	282 (85.5)	35.53	**<0** **.** **001**
Poorer	52 (32.5)	108 (67.5)		
Average	55 (34.4)	105 (65.6)		
Richer	22 (17.5)	104 (82.5)		
Richest	22 (22.9)	74 (77.1)		
Place lived in				
Rural	53 (27.2)	142 (72.8)	10.912	**0** **.** **004**
Urban	96 (25.7)	277 (74.3)		
Peri-urban	50 (16.4)	254 (83.6)		
Autonomy level				
Low	50 (18.2)	225 (81.8)	49.436	**<0** **.** **001**
Medium	46 (14.3)	275 (85.7)		
High	103 (37.3)	173 (62.7)		

Bold values refer to variables high statistical significance.

Table 5 describes the factors associated with IPV experiences among young women across selected states in Nigeria. Young women with primary or no formal education had five times odds of experiencing IPV compared to those with tertiary education (AOR = 4.284, *p* ≤ 0.001). Also, young women with secondary education had approximately three times higher odds of experiencing IPV compared to those with tertiary education (AOR = 2.512, *p* = 0.010). Young women whose households were located in rural communities had approximately three times odds of experiencing IPV compared to those in peri-urban communities (AOR = 2.534, *p* = 0.002). Also, young women in urban communities had more than three times odds of experiencing IPV compared to their counterparts in peri-urban communities (AOR = 3.359, *p* ≤ 0.001).

**Table 5:** Factors associated with intimate partner violence experiences among young women across selected states in Nigeria.

Characteristics	Adjusted Odds Ratio	95% Confidence Interval		*p*-value
		Lower	Upper	
Age (Years)				
<20	1.285	0.761	2.172	0.348
20–21	1.285	0.841	1.965	0.247
≥22	Ref			
Woman's level of education				
Primary or none	4.284	1.932	9.501	**<0** **.** **001**
Secondary	2.512	1.250	5.048	**0** **.** **010**
Tertiary	Ref			
Employment status				
Professional/Technical/Managerial	0.541	0.251	1.166	0.117
Sales and service	0.378	0.226	0.634	**<0** **.** **001**
Skilled manual	0.231	0.122	0.437	**<0** **.** **001**
Other	0.419	0.199	0.882	**0** **.** **022**
Unemployed	Ref			
Husband's level of education				
Primary or none	1.063	0.500	2.259	0.874
Secondary	1.459	0.844	2.523	0.177
Tertiary	Ref			
Husband's employment status				
Professional/Technical/Managerial	1.308	0.550	3.108	0.544
Sales and service	1.620	0.700	3.746	0.260
Skilled manual	1.556	0.661	3.662	0.311
Other	0.839	0.339	2.073	0.703
Unemployed	Ref			
Household wealth status				
Poorest	Ref			
Poorer	1.728	0.968	3.083	0.064
Average	2.530	1.426	4.490	**0** **.** **002**
Richer	0.902	0.461	1.764	0.783
Richest	1.560	0.795	3.059	0.196
Autonomy level				
Low	Ref			
Medium	0.627	0.369	1.067	0.085
High	2.323	1.419	3.801	**0** **.** **001**
Place lived in				
Rural	2.534	1.427	4.501	**0** **.** **002**
Urban	3.359	1.924	5.865	**<0** **.** **001**
Peri-urban	Ref			

Bold values refer to variables high statistical significance.

## 4 Discussion

This study examined the prevalence and factors associated with IPV among young married women in selected states in Nigeria. It revealed a substantial burden of IPV among young married women in selected states in Nigeria, with more than one in four respondents reporting at least one form of IPV. This prevalence is comparable to estimates reported in the Nigeria Demographic and Health Survey (NDHS), which documented that approximately 30% of ever-married women have experienced physical, sexual, or emotional violence from a spouse (16). Studies conducted among young and cohabiting young women in different geopolitical zones of Nigeria, similarly, report IPV prevalence ranging between 20% and 40% (20–22), depending on the population studied and the form of violence measured. The consistency of these findings highlights that IPV remains a persistent public health and human rights concern across diverse Nigerian contexts. Beyond Nigeria, similar prevalence levels have been documented across sub-Saharan Africa using nationally representative data. For example, a cross-sectional analysis of Demographic and Health Survey (DHS) data from Rwanda (23) reported IPV prevalence estimates was 37.7%. Similarly, a comparative study drawing on DHS datasets in Tajiskistan (24) found consistently high levels of IPV across diverse settings. The methodological similarity, that is, use of standardized DHS instruments and multivariate logistic regression, enhances comparability with the present study. The convergence of findings likely reflects shared structural drivers, including patriarchal norms, early marriage, and economic dependence, while differences in prevalence may be explained by variation in reporting norms, cultural acceptance of violence, and differences in measurement (e.g., lifetime vs. recent IPV).

Emotional violence was the most prevalent form, consistent with evidence that non-physical forms of abuse are often more widespread but less visible among young women in marital unions (16, 25). The predominance of emotional violence, particularly economic deprivation and verbal humiliation, highlights the everyday power asymmetries that characterize young women's marital relationships in many Nigerian settings. Nigerian qualitative studies have documented that emotional abuse, including verbal humiliation, economic restriction, and controlling behaviors, is often normalized within marriage and may not be readily recognized as violence (26, 27). In patriarchal settings where male authority is socially sanctioned, such non-physical forms of abuse may be perceived as disciplinary or corrective rather than abusive, contributing to their high prevalence and underreporting in formal systems (27). Comparable patterns have been reported in a study from Tanzania, which identified emotional and psychological abuse as highly prevalent but less frequently reported forms of IPV (28). The consistency across these contexts likely reflects the normalization of non-physical violence within patriarchal systems. However, differences in reported prevalence may stem from methodological variation, as facility-based studies often capture more severe cases, whereas population-based surveys, such as DHS, better capture the broader spectrum of IPV, including less visible forms like emotional abuse.

At the multivariate level, women's educational attainment emerged as one of the strongest predictors of IPV. Young women with primary or no formal education had more than four times higher odds of experiencing IPV compared with those who had tertiary education, while those with secondary education also faced significantly elevated risk. This finding aligns with several Nigerian studies and national surveys that consistently show education as a protective factor against IPV (16, 29–31). For example, analyses of NDHS data consistently demonstrate a graded relationship between women's educational attainment and IPV, with women who have completed secondary or higher education reporting lower lifetime exposure to spousal violence compared with those with no formal schooling (30, 31). Regional studies from Lagos, Ibadan, Kano, and Enugu, Nigeria have similarly shown that women with limited education are more likely to justify wife beating under certain circumstances, reinforcing the intergenerational transmission of norms that tolerate violence (32–34). Higher education may enhance women's awareness of their rights, improve communication and negotiation skills within marriage, and increase access to social and economic resources that reduce tolerance for abusive relationships. Conversely, limited education may reinforce dependence on husbands and acceptance of patriarchal norms that justify wife abuse. This protective role of education is consistently observed across multiple African settings. For instance, a DHS-based analysis of Tajikistan demonstrated a strong inverse association between women's educational attainment and IPV using multivariate logistic regression models (24). The consistency with the present study likely reflects common mechanisms, including delayed age at marriage, increased economic opportunities, and reduced acceptance of gender-based violence. Differences in the magnitude of association may reflect contextual variation in labor market access and the extent to which educational attainment translates into actual decision-making power.

Place of residence was also significantly associated with IPV experiences. Young women living in rural and urban communities had significantly higher odds of experiencing IPV compared with those in peri-urban areas. This pattern suggests that peri-urban settings may offer a relative protective effect, possibly due to a combination of greater exposure to modern gender norms, better access to information and services, and weaker enforcement of traditional practices compared with rural areas, while avoiding some of the economic stressors and overcrowding common in urban centers. Similar residential differentials have been reported in previous Nigerian and sub-Saharan African studies, though the direction and magnitude of effects often vary by context (35, 36). In Nigeria, rural residence has frequently been associated with higher acceptance of wife beating and limited access to formal support services, including legal protection and psychosocial care (37, 38). However, some urban-based studies have reported elevated IPV linked to unemployment, overcrowding, and economic stress, suggesting that urbanization alone does not confer protection (18, 29). The present findings contribute to this nuanced understanding by highlighting peri-urban residence as a potentially distinct social space with unique protective dynamics. While rural residence is often linked to stronger adherence to traditional gender norms and limited access to support services, urban settings may introduce economic stressors that increase IPV risk. The peri-urban protective effect observed in this study may reflect a transitional environment characterized by greater exposure to modern norms alongside relatively lower structural stress. Cross-country differences likely arise from variation in urbanization patterns, infrastructure, and access to social services.

Household wealth status also showed an independent association with IPV, with young women from households of average wealth having significantly higher odds of IPV compared with those from the poorest households. This finding contrasts with the commonly reported inverse relationship between wealth and IPV but is not entirely unexpected. Some studies suggest that transitions in economic status, rather than absolute poverty, may provoke conflict as traditional gender roles are re-negotiated, particularly among young couples (39, 40). In such contexts, economic strain combined with shifting expectations may heighten tensions and increase the risk of violence. Nigerian studies have similarly reported mixed associations between wealth and IPV (15, 41, 42). While extreme poverty is often linked to violence through economic stress pathways, some evidence suggests that households experiencing upward mobility or financial instability may experience increased conflict, particularly where shifts in economic contribution challenge traditional male breadwinner norms (43, 44). Similar non-linear associations between household wealth and IPV have been reported in DHS-based cross-country analyses (24), including studies from sub-Saharan African countries such as Malawi and Zambia. These studies, which employ wealth indices and multilevel regression models, suggest that relative economic change or instability may be more strongly associated with IPV than absolute poverty. The similarity in findings across contexts likely reflects tensions arising from shifting economic roles within households, while differences may depend on the stability of income sources and the degree to which economic changes are accompanied by shifts in gender norms.

Autonomy in household decision-making was significantly associated with IPV, with young women reporting high autonomy having higher odds of experiencing violence than those with low autonomy. This finding suggests that, in this context, autonomy may reflect contested power rather than protection. Among young married women, greater involvement in household decisions may challenge entrenched gender norms and expectations of male authority, thereby increasing the risk of conflict and violent backlash (26, 45). Similar patterns have been reported in other Nigerian and sub-Saharan African studies, where women's empowerment within strongly patriarchal settings has not consistently translated into reduced IPV risk (26, 46).

Analyses of Nigerian survey data have demonstrated that women's participation in household decision-making does not uniformly reduce IPV risk (15, 16) and, in some contexts, may be associated with increased exposure when empowerment occurs in environments where patriarchal norms remain rigid. This phenomenon has been described as “male backlash,” whereby shifts in perceived gender hierarchy provoke attempts to reassert control through violence (46). This phenomenon has been described as “male backlash,” whereby shifts in perceived gender hierarchy provoke attempts to reassert control through violence (46). This interpretation is consistent with theoretical perspectives on gender and empowerment, who conceptualizes empowerment as a dynamic and context-dependent process involving resources, agency, and achievements (26, 46). Empirical evidence from DHS-based multi-country analyses in sub-Saharan Africa (47, 48) similarly demonstrates that women's decision-making autonomy is not uniformly protective against IPV. These studies typically employ cross-sectional designs and multivariate regression models, allowing for comparison with the present findings. The consistency of this pattern suggests that increases in women's agency may initially disrupt entrenched gender norms in contexts where normative change lags behind behavioral change. Differences across settings may reflect variation in how autonomy is operationalized, the stage of gender norm transition, and the extent to which male attitudes evolve alongside women's empowerment. Thus, the positive association observed in this study likely reflects transitional gender dynamics rather than a direct causal effect of autonomy.

Notably, age at marriage and number of children were not significant predictors of IPV after adjusting for other factors, despite showing associations at the bivariate level. This suggests that structural and contextual factors, such as education, residence, wealth, and autonomy, play a more decisive role in shaping IPV risk among young married women than age alone. Although early marriage remains prevalent in parts of Nigeria, particularly in northern regions, previous Nigerian studies have shown that its association with IPV is often mediated by education, economic dependency, and autonomy (18, 25). Once these structural factors are controlled for, the independent effect of age at marriage may diminish, as observed in the present study.

## 5 Limitations

This study had a few limitations. First, the cross-sectional design precludes causal inference, and the observed associations should be interpreted as correlational rather than causal. Second, IPV was self-reported and may have been underreported due to recall bias or social desirability bias, particularly for sensitive forms such as sexual violence. Although confidentiality was emphasized, stigma and fear of repercussions may have influenced disclosure. Third, the study did not collect information on husbands' age or the age difference between partners. Age disparity within marital unions has been identified in previous literature as an important determinant of intimate partner violence and household power dynamics. In the study setting, questions regarding husbands' age were perceived as sensitive, and preliminary field experience suggested potential reluctance to disclose such information. As a result, this variable was not included in the final instrument. The omission limited our ability to assess the role of partner age difference in shaping autonomy and IPV risk among young married women.

Despite these limitations, this study has notable methodological and contextual strengths. The relatively large sample of young married women enhances statistical power and the precision of estimates. The use of standardized IPV measures aligned with nationally and internationally validated survey instruments improves comparability with existing Nigerian and global evidence. By focusing specifically on young married women, a group often underrepresented in IPV research despite heightened vulnerability due to early marriage, economic dependence, and constrained autonomy, the study provides age-specific insights that are critical for targeted intervention design. Furthermore, the inclusion of multiple states and diverse residential settings (rural, urban, and peri-urban) strengthens the contextual relevance of the findings and allows for examination of geographic differentials in IPV risk. Collectively, these strengths enhance the robustness, policy relevance, and practical applicability of the study's findings within the Nigerian context.

## 6 Conclusion

The findings indicate that women's educational attainment, place of residence, and household socioeconomic context are key determinants of IPV among young married women in Nigeria. Interventions should therefore prioritize structural and context-specific strategies. First, strengthening girls' and young women's access to secondary and tertiary education should be a central IPV prevention strategy. Federal and State Ministries of Education, in collaboration with social welfare agencies, should implement policies that prevent school discontinuation due to early marriage and promote re-entry pathways for married young women. Educational advancement not only improves economic independence but may also shift norms that tolerate violence.

Second, given the elevated IPV risk observed among women residing in rural and urban communities compared with peri-urban areas, geographically tailored interventions are warranted. In rural areas, expanding access to legal protection, community-based reporting mechanisms, and gender-transformative programming is critical. In urban settings, interventions should address economic stressors, unemployment, and overcrowding that may exacerbate household conflict. State Ministries of Women Affairs and community-based organizations should design locality-specific prevention strategies rather than adopting uniform statewide approaches.

Third, the association between household wealth and IPV underscores the need for economic stabilization and social protection measures targeted at young couples. Conditional cash transfer programs, livelihood support schemes, and employment initiatives for young households may help mitigate financial strain and reduce conflict linked to economic insecurity. However, economic empowerment initiatives should be paired with gender norm–transformative components to prevent potential backlash associated with shifting power dynamics.

Finally, the observed association between higher autonomy and increased IPV risk suggests that empowerment interventions must be carefully contextualized. Programs promoting women's decision-making power should simultaneously engage men and boys to address rigid patriarchal norms and reduce resistance to changing gender roles. Without such complementary strategies, empowerment efforts alone may unintentionally heighten conflict. Also, future studies should include husband's age or spousal age difference in their data collection instruments and analyses, as it affects power dynamics within marriage.

## Data Availability

The original contributions presented in the study are included in the article/Supplementary Material, further inquiries can be directed to the corresponding author.
